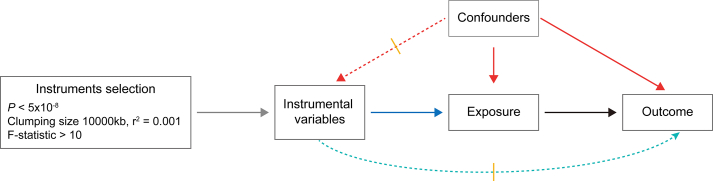# Mendelian randomization demonstrates a causal link between peripheral circulating acylcarnitines and intracranial aneurysms

**DOI:** 10.1016/j.neurot.2024.e00428

**Published:** 2024-08-03

**Authors:** Ying Wang, Kang Xie, Junyu Wang, Fenghua Chen, Xi Li, Longbo Zhang

**Affiliations:** a Department of Neurosurgery, National Clinical Research Center of Geriatric Disorders, Xiangya Hospital, Central South University, Changsha, 410008, China; b Research Center for Cerebrovascular Disease, Central South University, Changsha, 410008, China; c Department of Clinical Pharmacology, Xiangya Hospital, Central South University, 87 Xiangya Road, Changsha, Hunan, 410008, China; d Institute of Clinical Pharmacology, Central South University, Hunan Key Laboratory of Pharmacogenetics, Changsha, Hunan, 410008, China; e Departments of Neurosurgery, Changde Hospital, Xiangya School of Medicine, Central South University, 818 Renmin Street, Wuling District, Changde, Hunan 415003, China; f Department of Neurosurgery, National Clinical Research Center of Geriatric Disorders, Research Center for Cerebrovascular Disease, Xiangya Hospital, Central South University, Changsha, 410008, China

**Keywords:** Intracranial aneurysm, Mendelian randomization, Acylcarnitines, Hypertension

## Abstract

Intracranial aneurysm (IA) is the most prevalent type of cerebral vascular disease causing life-threatening subarachnoid hemorrhages (SAH). A long-term vascular structure remodeling is considered as the main pathophysiological feature of IAs. However, the causal factors triggering the pathophysiological process are not clear. Recently, the abnormalities of peripheral circulating proteins and metabolites have been found in IAs patients and associated with the ruptures. We comprehensively investigated the potential causal relationship between blood metabolites and proteins and IAs using the mendelian randomization (MR) analysis. We applied two-sample MR to explore the potential causal association between peripheral circulating metabolites (191 blood metabolites) and proteins (1398 proteins) and IAs using data from the FinnGen study and the GWAS datasets published by Bakker et al. We identified palmitoylcarnitine, stearoylcarnitine and 2-tetradecenoylcarnitine as causal contributors of IAs and ruptures. Further two-step mediation MR analysis suggested that hypertension as one of the contributors of IAs and ruptures mediated the causal relationship between palmitoylcarnitine, stearoylcarnitine and 2-tetradecenoylcarnitine and IAs. Together, our study demonstrates that blood metabolic palmitoylcarnitine, stearoylcarnitine and 2-tetradecenoylcarnitine are causally linked to the formation and rupture of IAs. Hypertension partially mediates the causal effects.

## Introduction

Intracranial aneurysms (IAs), the most common type of cerebral vascular disease, account for 85% of non-traumatic subarachnoid hemorrhages (SAH) [1]. Although recent development of radiographic diagnosis and treatment such as surgical clipping and intravascular interventions has dramatically improved the prognosis of IAs, aneurysmal SAH is still life-threatening with around 25% mortality rate [2,3]. IAs are out-pouching usually located at the outer curvatures or bifurcations of intracranial arterial walls. Endothelial injury or dysfunction, smooth muscle cells phenotypic switching and focal inflammatory cells accumulation are considered as the main features associated to the formation and rupture of IAs [4,5]. However, the mechanisms triggering above pathophysiological changes are not clear.

Recently, the abnormalities of peripheral circulating proteins and metabolites have been found in IAs patients and associated with the ruptures [[6], [7], [8]]. For example, the serum protein proinflammatory cytokines (IL-1β) and TNF-α were reported to be increased in IAs [9], while elevated D-dimer, cystatin C and C-reactive protein are significantly associated with IAs rupture [10]. In addition, a plasma metabolic profiling showed that the cotinine in serum were associated with IAs and acted as an independent risk factor of ruptures [11]. However, the causal links between blood metabolites and proteins and IAs remain unknow. Mendelian randomization (MR) analysis is a well-characterized method that utilizes single-nucleotide polymorphisms (SNPs) as genetic instrumental variables (IVs) to investigate the causal relationship between a given exposure and an outcome [12]. This approach is grounded in Mendel's laws of segregation and independent assortment, where genetic alleles are randomly assorted during meiosis independently of environment and other genetic factors, with the exception of linkage disequilibrium. Unlike observational studies that are susceptible to confounding, MR functions similarly to a randomized controlled trial, thereby strengthening causal inference between exposure and outcome with reverse causality prevented because germline genotype cannot be modified by disease [13]. The advancement of genome-wide association studies (GWAS) has significantly facilitated MR studies. GWAS can examine millions of SNPs to determine their associations with various traits, thus providing a rich resource of genetic instruments for MR studies. This development allows for more robust and comprehensive exploration of genetic influences on complex traits, facilitating the identification of potential causal pathways and deepening our understanding of the genetic basis of diseases and other outcomes [14].

Thus, in this study, to identify the causal relationship between blood metabolites and proteins and IAs, we applied MR using four published GWAS datasets from Shin et al. [15], Sun et al. [16], Bakker et al. [17] and FinnGen [18]. FinnGen is an ongoing project studying the genome and national health of 500,000 Finnish biobank participants, leveraging the advantage of population isolates, which has released GWAS data for various traits [18].

## Methods

### Study design

**Fig. 1:**
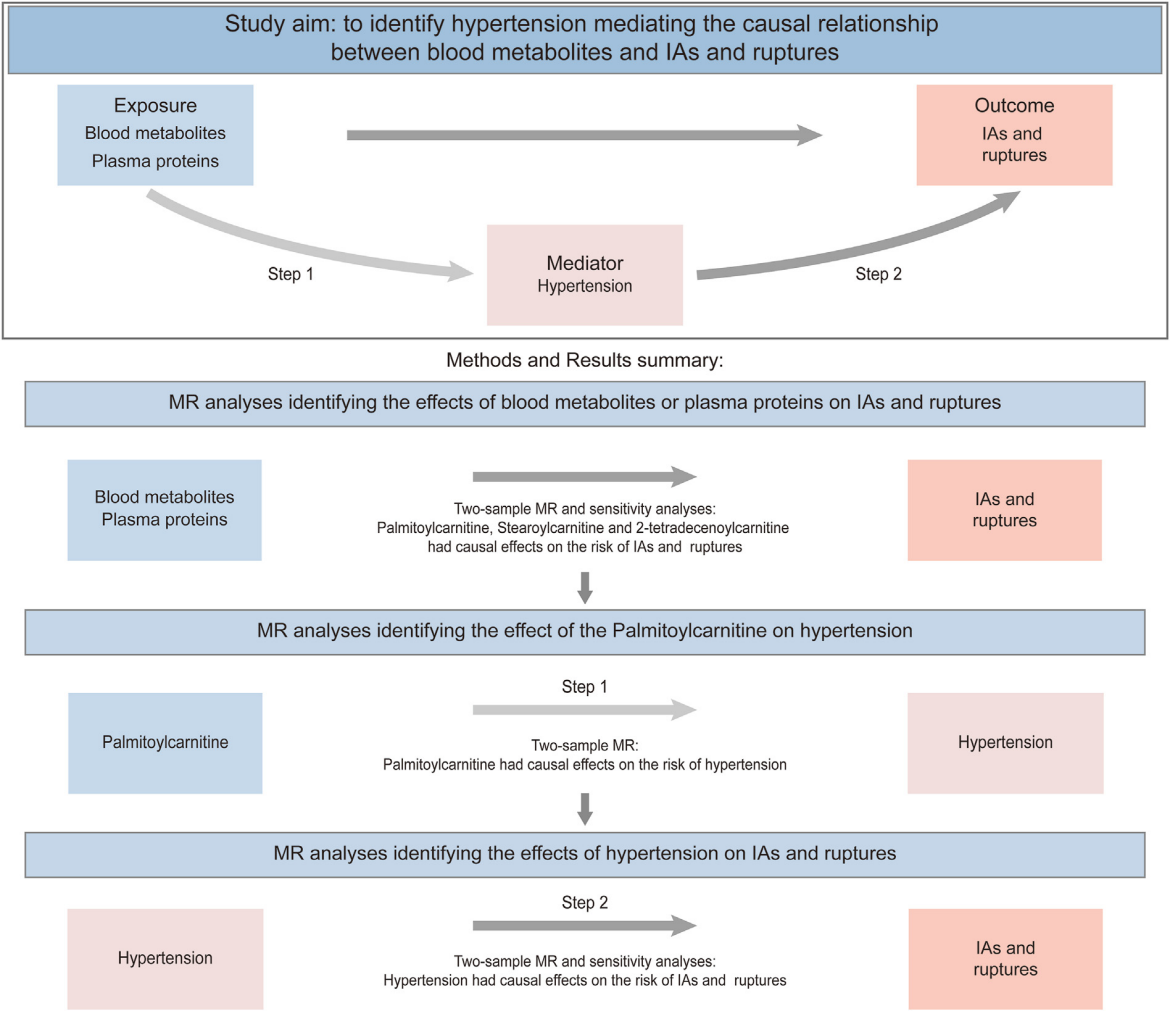
**The illustration of mendelian randomization (MR) study.** We performed two-sample MR to investigate the causal links between exposures (blood metabolites and plasma proteins) and outcomes (IAs and ruptures), and two-step MR to identify the hypertension as a mediator of the causal association between blood metabolites and IAs (Step 1 and 2).

This study followed the Strengthening the Reporting of Observational Studies in epidemiology using mendelian randomization (STROBE-MR) guideline [19]. We systematically assessed the causal relationship between exposures (blood metabolites or plasma proteins) and outcomes (IAs and ruptures). The MR analyses were based on the following IV assumptions (core assumptions in MR studies): 1) relevance (IV is associated with the exposure); 2) independence (IV share no unmeasured cause with the outcome); 3) exclusion restriction (IV doesn't affect the outcome except potentially via the exposure) [20]. The first assumption can be directly evaluated by examining the strength of the association between IV and the exposure of interest. However, the second and third assumptions cannot be empirically proven, which necessitate both judgment by the investigators and the implementation of various sensitivity analyses [21]. In this study, the genetic information for IAs was sourced from two GWAS datasets (published by Bakker et al. and FinnGen). The study design was illustrated in Fig. 1. According to the original GWAS protocols, informed consents were obtained from all participants, and all necessary ethical approvals for the GWAS were secured by the original GWAS authors.

### GWAS data for exposure, mediation, and outcome

The blood metabolites in either plasma or serum of this study were based on a GWAS of 7824 adult individuals from 2 European populations (Table S1) [15]. A subset of 486 metabolites, consisting of 309 known and 177 unknown metabolites, was available for genetic analysis. The 309 blood metabolites included eight metabolic subgroups (amino acids, carbohydrates, cofactors and vitamins, energy, lipids, nucleotides, peptides, and xenobiotic metabolism) followed by the definition of the Kyoto Encyclopedia of Genes and Genomes (KEGG) database [22]. The GWAS of plasma proteins (plasma pQTL data) were retrieved from the INTERVAL study, which quantified 3622 plasma proteins in 3301 healthy participants (Table S1) [16]. For mediation GWAS data, we obtained genetic estimates for hypertension from the FinnGen, including 111,581 cases and 265,626 controls (Table S1) [18]. The GWAS datasets of IAs were obtained from Bakker et al. and FinnGen. The cohort from Bakker et al. included 7495 IAs (5425 ruptured and 2070 unruptured) and 71,934 controls [17]. Summary statistics obtained from FinnGen included 7924 IAs (5342 ruptured and 2582 unruptured) and 342,673 controls (Table S1) [18]. We imported the aforementioned GWAS datasets into R for data processing and cleaning. All data were stored in data frames.

## Mendelian Randomization

### Genetic instruments selection

MR employs genetic variants associated with exposure as IVs to investigate the potential causal relationship with an outcome. In this study, the genetic instruments for exposures were rigorously selected using stringent criteria, including genome-wide statistical significance (*P* ​< ​5 ​× ​10⁻⁸), and not in linkage disequilibrium (LD) with other SNPs (r^2^ ​< ​0.001, within a clumping window of 10,000 ​kb). In addition, F-statistic was employed to assess the strength of the genetic instruments for exposure (Table S8). Formula F = ((N - K - 1)/K) ​× ​((R^2^/(1 - R^2^)) was applied to compute the F-statistic values (N represents the sample size of the exposure GWAS data, K represents the total number of SNPs included in each MR analysis, and R^2^ signifies the explained variance of genetic variation). A threshold of 10 was set to distinguish strong and weak instruments, with a higher F-statistic indicating a more robust instrument [23,24].

### Combining exposure and outcome

Exposures and outcomes were merged based on shared SNPs and harmonized using the “harmonise_data” function in the R package “TwoSampleMR” [25]. The combined datasets included the associations between metabolites and IAs, proteins and IAs, metabolites and hypertension, and hypertension and IAs. These combined datasets were then used for the subsequent MR analysis. In addition, to verify the metabolites or proteins with causal relationships identified using the GWAS data from Bakker et al. as the outcome, we selected the metabolites and proteins shared in the combined data for further analysis. Ultimately, 191 metabolites and 1398 proteins were used as exposures for analysis. All exposure instrumental variables utilized in this study are listed in Table S9.

### Statistical analysis

All MR analyses were performed using the “TwoSampleMR” package [25] in R version 4.3.1. Two-sample MR framework incorporating the sensitivity analyses were utilized for both primary MR (metabolites or proteins → IAs and ruptures), and two-step MR (step 1: metabolites → hypertension; step 2: hypertension → IAs and ruptures). In the main analysis, the Wald ratio method was performed to calculate MR estimates for each SNP. These SNP estimates were subsequently subjected to meta-analysis using inverse variance weighted (IVW). MR-Egger, Weighted median, Simple mode, and Weighted mode were used to meta-analyze the SNP estimates. In cases where the proposed instruments consisted of more than two variants, MR-Egger regression was conducted to address potential pleiotropy in the relationship between exposure of interest and outcomes based on Egger intercepts [26]. To assess heterogeneity among genetic instruments, Cochran's Q test was employed to capture heterogeneity (*P* ​< ​0.05 and I^2^ ​> ​25%) [27,28]. When significant heterogeneity was detected, a random-effect IVW model was applied; in contrast, a fixed-effect IVW model was employed in such situations. Multiple testing-adjusted significance thresholds for the sensitivity analyses were established using Bonferroni corrections. When 191 blood metabolites served as exposures, *P*-values below 2.62 ​× ​10^−4^ (*P* ​= ​0.05/191 blood metabolites) were defined as significant. For 3 blood metabolites, *P*-values below 0.0167 (*P* ​= ​0.05/3 blood metabolites) were defined as significant. For 1398 plasma proteins, *P*-values below 3.58 ​× ​10^−5^ (*P* ​= ​0.05/1398 plasma proteins) were defined as significant.

A mediation analysis was performed to evaluate the contribution of hypertension to the causal association between metabolites and IAs. The total effect of exposure on outcome encompasses both direct effect and indirect effect mediated through one or more intermediaries. In this study, the total effect is captured through a standard univariable MR analysis which corresponds to the primary MR. To differentiate direct and indirect effect, a two-step MR approach was utilized. The Product of coefficient method was applied to estimate the beta of indirect effect while the Delta method was applied to estimate standard error (SE) and confidence interval (CI) [29,30]. The accuracy of all causal association hypotheses between exposures and potential outcomes was assessed by the MR Steiger directionality test (Table S8).

## Results

### Blood metabolites, but not plasma proteins act as causal contributor to IAs

**Fig. 2:**
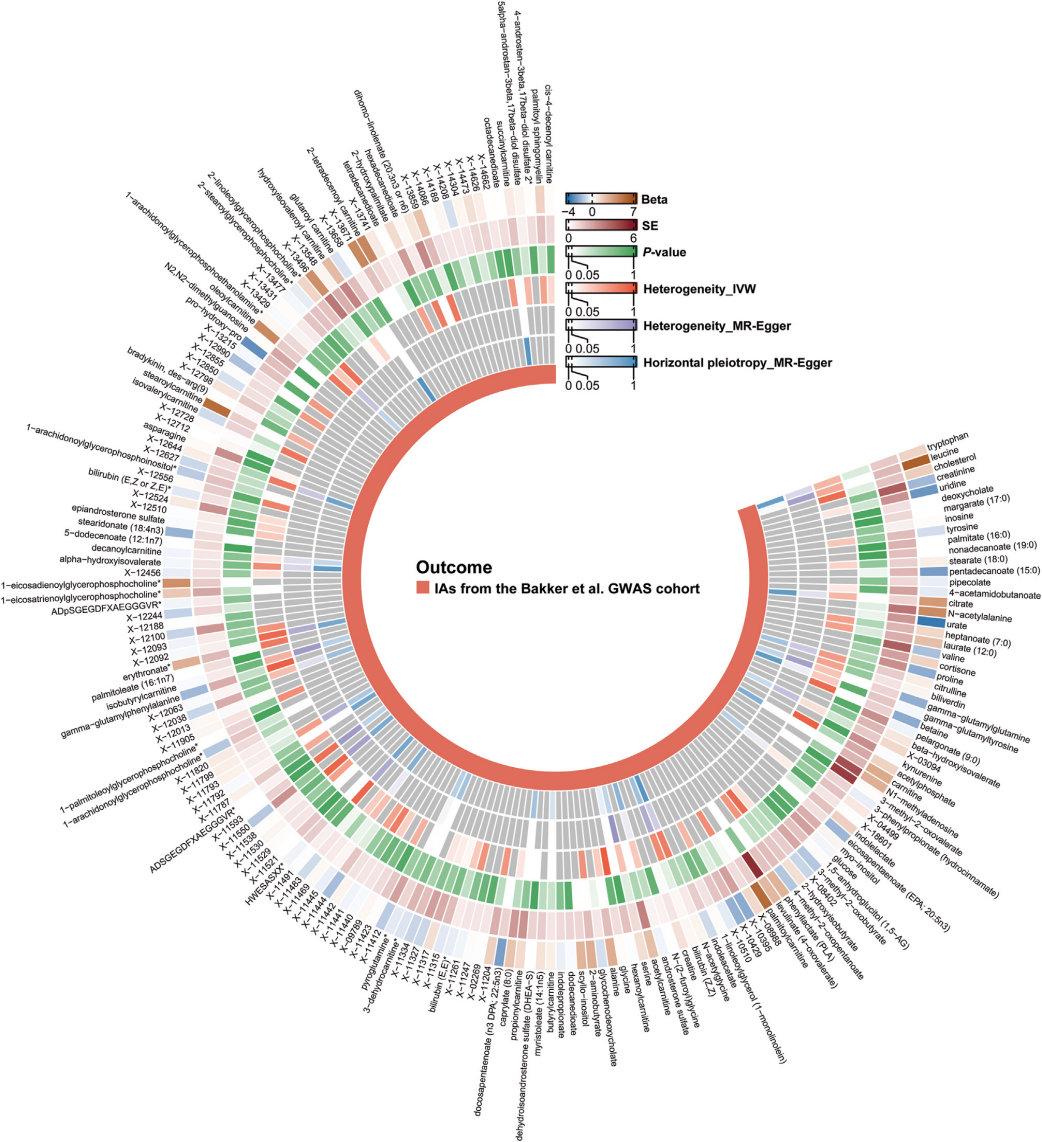
**Circular heatmap indicates the results of the MR, pleiotropy and heterogeneity analysis.** The circulars indicate the results of MR analysis (from outside to inside): Beta, standard error (SE), *P* value of Wald Ratio or IVW method, *P* values of heterogeneity (IVW and MR-Egger) and horizontal pleiotropy.

**Fig. 3:**
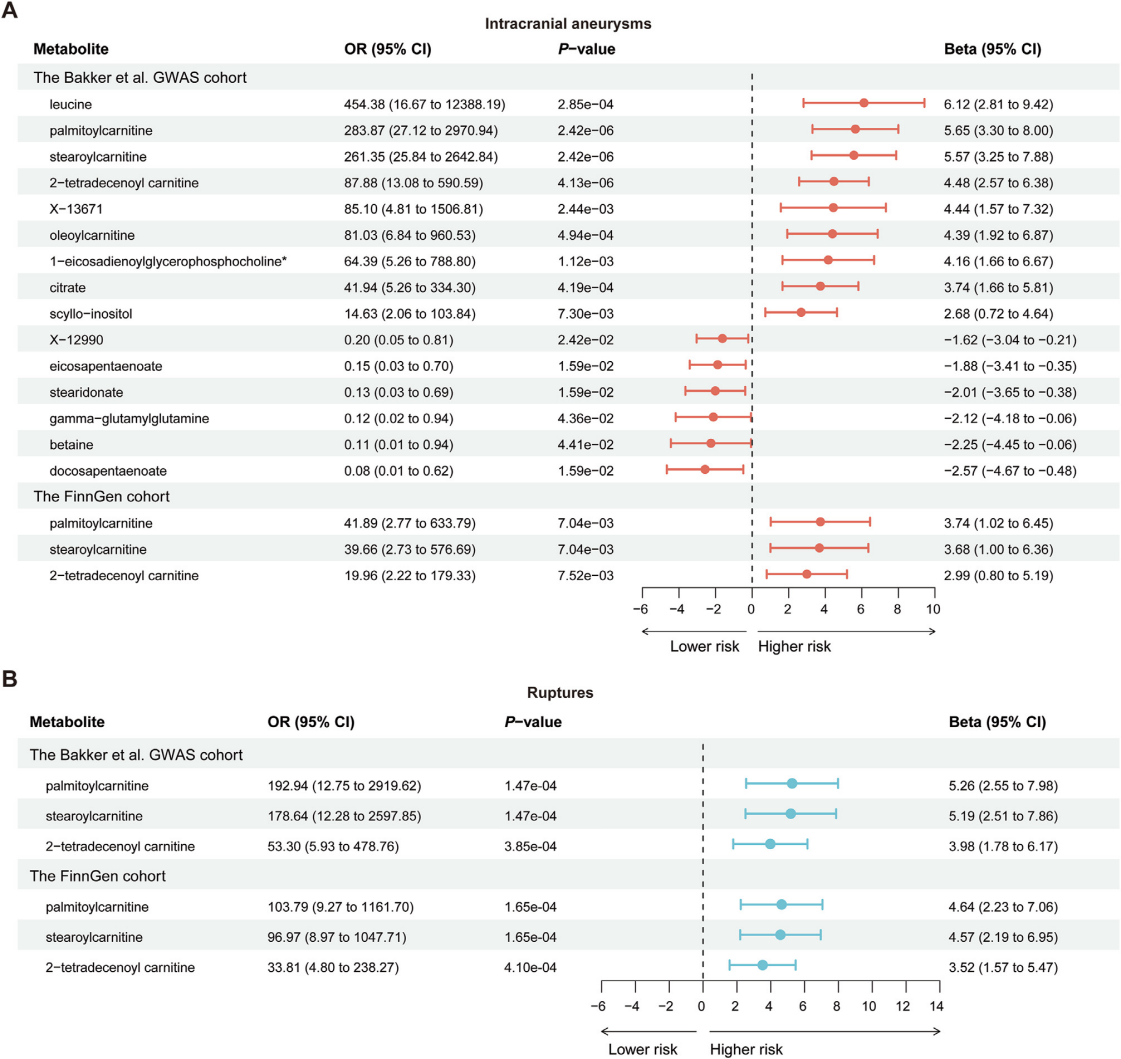
**Forest plots for two-sample MR analysis. A:** Forest plot for MR results between blood metabolites and IAs using the Bakker et al. GWAS cohort and FinnGen cohort. **B:** Forest plot represents the MR results between blood metabolites and ruptured IAs in the Bakker et al. GWAS cohort and FinnGen cohort. The dots indicate the causal estimates on the Beta scale, and the whiskers depict the 95% confidence intervals (CI).

IAs form and rupture due to a combination of genetic and environmental factors. The main issue in IAs is blood vessel remodeling, which is linked to oxidative stress and inflammation [31,32]. Studies show that blood metabolites [[33], [34], [35]] and plasma proteins, such as cytokines, growth factors, and matrix metalloproteinases [[36], [37], [38]], are involved in these processes. Here, to comprehensively investigate the impacts of peripheral circulating metabolites and proteins on IAs formation, we performed two-sample MR analysis parallelly using blood metabolites [15] and plasma proteins [16] as exposures while IAs as outcome. We firstly utilized the IAs GWAS datasets obtained from Bakker et al., comprising 7495 IAs patients and 71,934 controls, as the outcome, and employed Shin et al. GWAS dataset of blood metabolites as exposures to compute the MR effect for SNP (Wald ratio: SNP n ​= ​1; IVW: SNP n ​> ​1) (Fig. 2, Table S2). A total of 15 potential causal metabolites were primarily identified (*P* ​< ​0.05) (Fig. 3A). After multiple-testing correction, we eventually identified three acylcarnitine metabolites palmitoylcarnitine, stearoylcarnitine and 2-tetradecenoylcarnitine as the causal exposures of IAs outcome (*P* ​< ​2.62 ​× ​10^−4^) (Fig. 3A). To validate our findings, we used another GWAS summary data from the FinnGen including 7924 IAs patients and 34,2673 controls as the outcome. The results showed that all three blood metabolites we identified in the Bakker et al. GWAS cohort were significantly beyond the stringent Bonferroni threshold (*P* ​< ​0.0167) with the same direction of effect (Fig. 3A, Table S4). As sensitivity analysis necessitates two or more SNPs, it was not conducted for these three metabolites (Fig. 2, Tables S2 and 9). In addition, the same method was applied to determine the potential causal relationship between plasma proteins and IAs in Bakker et al. GWAS datasets by setting 1398 plasma proteins as exposures. However, no significant plasma proteins reached the suggestive threshold (*P* ​< ​3.58 ​× ​10^−5^) in either IVW or Wald ratio method (Table S3).

### Palmitoylcarnitine, stearoylcarnitine and 2-tetradecenoylcarnitine are casually associated with IAs rupture

Aneurysmal SAH is a life-threatening stroke [39]. Therefore, we further evaluated whether the three acylcarnitine metabolites palmitoylcarnitine, stearoylcarnitine and 2-tetradecenoylcarnitine are casually associated with IAs rupture and aneurysmal SAH. Within the Bakker et al. GWAS cohort including 5425 ruptured IAs and 71,934 controls, we found that all three metabolites were causally associated with a high risk of ruptured IAs (*P* ​< ​0.0167) (Fig. 3B, Table S4). We then validated these 3 metabolites in the FinnGen cohort including 5342 ruptured IAs and 342,673 controls. Consistent with the Bakker et al. GWAS cohort, palmitoylcarnitine, stearoylcarnitine and 2-tetradecenoylcarnitine were identified as the causal factors of IAs associated SAH (*P* ​< ​0.0167) (Fig. 3B, Table S4). Accordingly, we identified that palmitoylcarnitine, stearoylcarnitine and 2-tetradecenoylcarnitine as the blood metabolic factors contributed to the formation and rupture of IAs.

### Palmitoylcarnitine, stearoylcarnitine and 2-tetradecenoylcarnitine contribute to IAs and aneurysmal SAH partially through hypertension

**Fig. 4:**
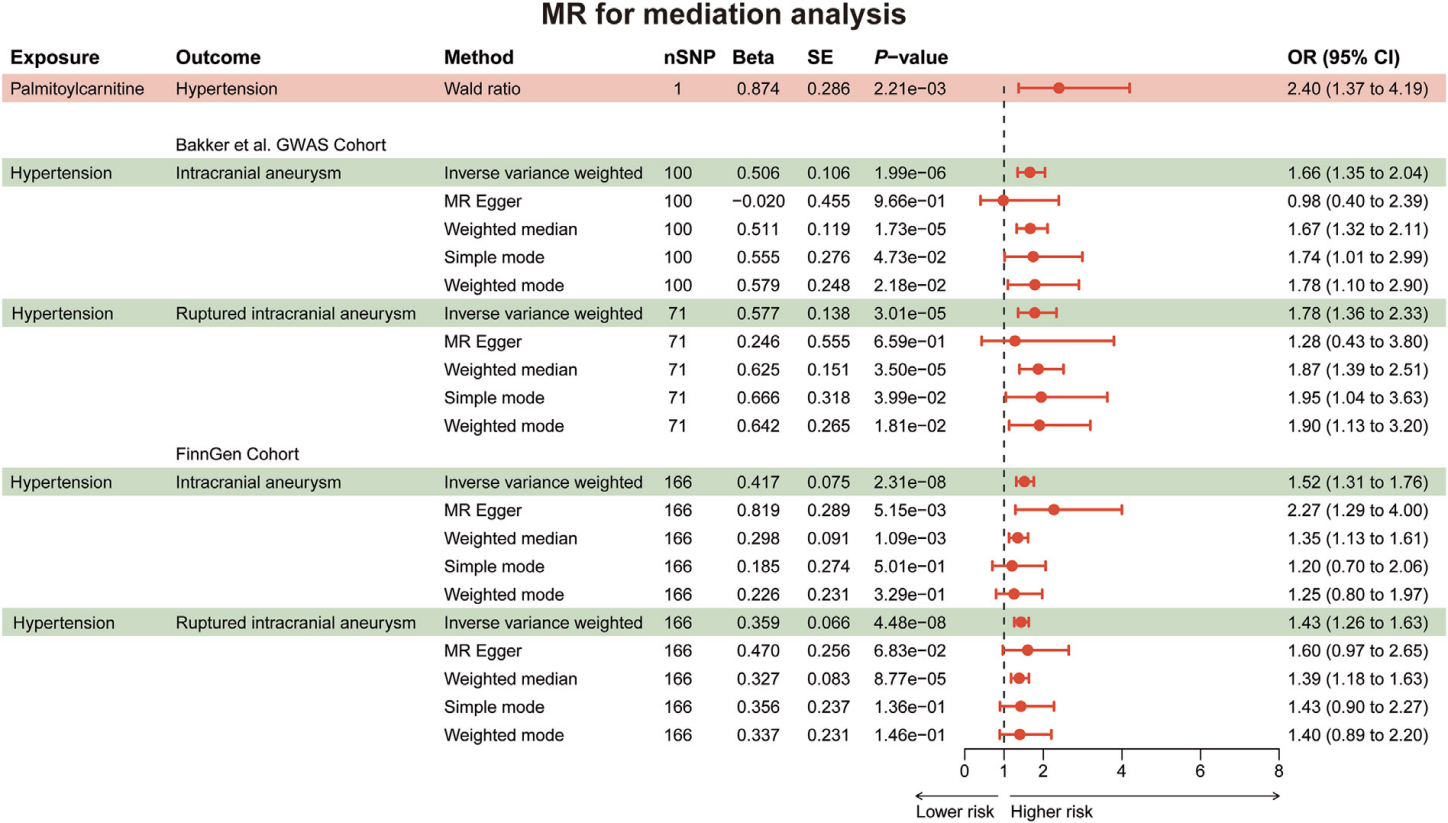
**Forest plots for two-step mediation MR.** Forest plots display MR results between palmitoylcarnitine and hypertension, and the results between hypertension and IAs and ruptures. The dots are the causal estimates on the OR scale, and the whiskers depict the 95% CI.

**Fig. 5:**
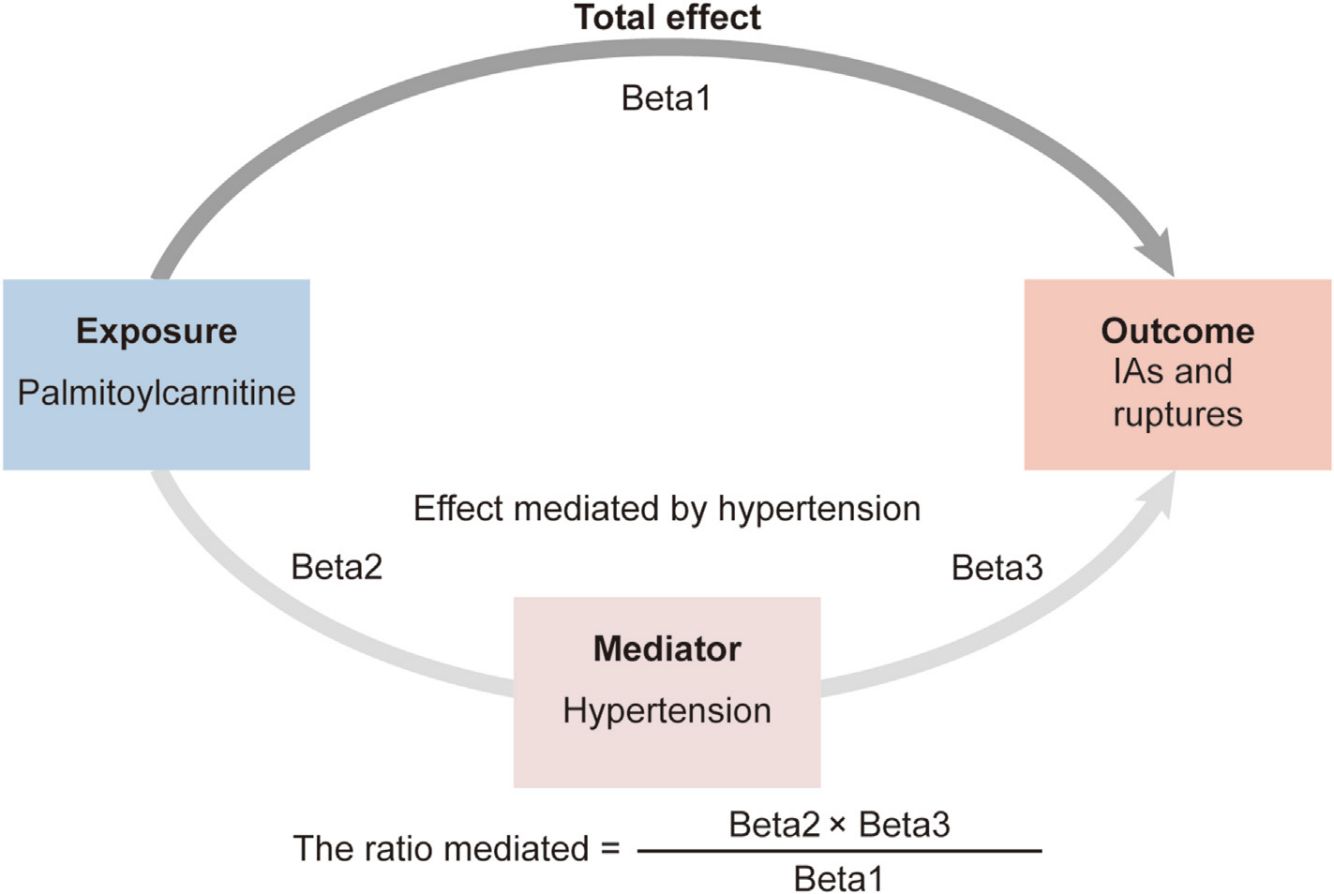
**The illustration of coefficient product method.** The dark grey arrow represents the total effect of palmitoylcarnitine on IAs and rutptures. The light grey arrow represents the effect of palmitoylcarnitine on IAs and ruptures mediated by hypertension. The equation of the coefficient product method is on the bottom. Beta1: the total impact of palmitoylcarnitine on outcomes (IAs and ruptures), Beta2: causal effect of palmitoylcarnitine on mediator (hypertension), Beta3: causal effect of hypertension on outcomes (IAs and ruptures).

Palmitoylcarnitine, stearoylcarnitine and 2-tetradecenoylcarnitine are involved in the transport and metabolism of fatty acid within cells [40], which are crucial for the cardiovascular health such as hypertension [[41], [42], [43]]. Of note, hypertension is a recognized high-risk factor for IAs and there is a large amount of studies suggesting that hypertension promotes IAs formation and rupture [31,[44], [45], [46], [47]]. Thus, to understand whether palmitoylcarnitine, stearoylcarnitine and 2-tetradecenoylcarnitine contributed to IAs and aneurysmal SAH through hypertension, we conducted two-step mediation MR analysis. We initiated the analysis by conducting a two-sample MR to assess the causal effects of palmitoylcarnitine, stearoylcarnitine, and 2-tetradecenoylcarnitine on hypertension. Notably, both palmitoylcarnitine and stearoylcarnitine are associated with the same variant (rs419291), while 2-tetradecenoylcarnitine is linked to another variant (rs270613); however, there exists a high degree of linkage disequilibrium between the two variants [48]. Given this, we randomly selected palmitoylcarnitine for further analysis. In the step 1 ​MR analysis, palmitoylcarnitine served as the exposure, and hypertension was the outcome. The MR results indicated that palmitoylcarnitine exhibited a causal effect on hypertension (OR [95% CI] ​= ​2.40 [1.37, 4.19], *P* ​= ​2.21 ​× ​10^−3^) (Fig. 4, Table S5). Subsequently, we proceeded to validate the effect of hypertension on IAs and rupture using step 2 ​MR analysis (Fig. 4, Table S5). In this step, hypertension was used as the exposure, and two IAs GWAS datasets (from Bakker et al. and FinnGen) were utilized as outcomes, respectively. Consistent with prior studies, the results demonstrated that hypertension was associated with a higher risk of IAs (OR [95% CI] ​= ​1.66 [1.35, 2.04], *P* ​= ​1.99 ​× ​10^−6^) and ruptures (OR [95% CI] ​= ​1.78 [1.36, 2.33], *P* ​= ​3.01 ​× ​10^−5^) in the Bakker et al. GWAS cohort, as well as in the FinnGen cohort (IAs: OR [95% CI] ​= ​1.60 [1.25, 2.05], *P* ​= ​1.80 ​× ​10^−4^; ruptures: OR [95% CI] ​= ​1.43 [1.26, 1.63], *P* ​= ​4.48 ​× ​10^−8^). A random-effect IVW model was applied to adjust for heterogeneity (Table S6). In addition, we calculated the indirect effect of palmitoylcarnitine on IAs and ruptures that can be accounted by hypertension (Fig. 5). The results showed that the proportion of hypertension mediated the effect of palmitoylcarnitine on the risk of IAs and ruptures were 7.8% and 9.6% (from the Bakker et al. GWAS cohort), and 9.8% and 6.8% (from the FinnGen cohort), respectively (Table S7). Altogether, our data suggested that palmitoylcarnitine, stearoylcarnitine and 2-tetradecenoylcarnitine contributed to IAs and aneurysmal SAH partially through hypertension.

## Discussion

Intracranial aneurysm is a life-threatening brain vascular disease caused by multiple systemic factors and focal vascular structure remodeling. In this study, we applied mendelian randomization analysis to identify the potential causal factors in blood proteins and metabolites. The results suggested that three peripheral circulating acylcarnitine metabolites (palmitoylcarnitine, stearoylcarnitine, 2-tetradecenoylcarnitine) causally contributed to IAs and ruptures. Acylcarnitine is an ester produced by the conjugation of fatty acids with l-carnitine [40], which transports acyl groups from cytoplasm to mitochondrial matrix for β oxidation, thus producing energy to maintain cell activities [49]. Palmitoylcarnitine, the most abundant long-chain acylcarnitine in plasma, is formed through the esterification of carnitine with palmitic acid, a 16-carbon saturated fatty acid [50,51]. This metabolite is a crucial intermediate for transporting long-chain fatty acids into the mitochondria for beta-oxidation [52]. Similarly, stearoylcarnitine, derived from stearic acid (an 18-carbon saturated fatty acid), plays a role in the mitochondrial transport of stearic acid [53]. Elevated levels of these metabolites have been associated with mitochondrial dysfunction and oxidative stress [40,53,54], both of which are critical in the development of vascular pathologies. Mitochondrial dysfunction in vascular smooth muscle cells and endothelial cells can lead to impaired cellular energy homeostasis and increased production of reactive oxygen species (ROS) [55,56]. ROS contribute to the degradation of the extracellular matrix and weakening of the vessel wall, predisposing individuals to IA formation [57]. Studies have demonstrated that palmitoylcarnitine and stearoylcarnitine can modulate inflammatory pathways by activating nuclear factor kappa B and other pro-inflammatory transcription factors [58,59]. Chronic inflammation within the arterial wall is a hallmark of IA development [31,60], suggesting that these acylcarnitines might play a direct role in exacerbating inflammatory responses that compromise vascular integrity. Additionally, 2-tetradecenoylcarnitine, an unsaturated long-chain acylcarnitines with a double bond in its 14-carbon chain, has been implicated in lipid peroxidation processes [40,61]. Lipid peroxidation products are known to have deleterious effects on cellular membranes and are potent inducers of inflammatory signaling pathways [62,63]. The presence of 2-tetradecenoylcarnitine can enhance lipid peroxidation, leading to further vascular damage and promoting the inflammatory milieu necessary for aneurysm progression. Our identification of these three acylcarnitines was in line with the recent understanding of IAs formation which involves endothelial dysfunction, mitochondrial dysfunction and smooth muscle cells phenotypic switching [64,65].

We next explored how these three acylcarnitine metabolites contribute to IAs and ruptures. Since acylcarnitine has been suggested to be highly involved in cardiovascular disease^65-68^, especially positively associated with increased blood pressure [[41], [42], [43],[66], [67], [68], [69], [70], [71]], meanwhile hypertension is a recognized high-risk factor for IAs promoting the processes of formation and rupture [2,31,[44], [45], [46], [47],[72], [73], [74], [75]], we thus performed a two-step MR to exam the causal links between these three acylcarnitines and IAs. The data indicated that the hypertension partially mediated the effects of palmitoylcarnitine, stearoylcarnitine, and 2-tetradecenylcarnitine on IAs and ruptures. Accordingly, the study indicated that metabolic palmitoylcarnitine, stearoylcarnitine and 2-tetradecenoylcarnitine are causally linked to the formation and rupture of IAs partially through hypertension.

There are several limitations in our study. The hypertension only partially accounted for the causal association between blood metabolites and IAs with a relatively low proportion. The identification of other mediations would be needed. Previous studies have shown that common risk factors for IAs include genetic factors (such as autosomal dominant polycystic kidney disease), smoking, sex, age, alcohol consumption, medication, and drug use [32]. However, the above-mentioned risk factors lack logical coherence in exposure and outcome in MR studies, or there is no corresponding GWAS data to support effective MR analysis. Therefore, further MR studies and exploration of mediators depend on future research into the mechanisms related to IA and the ongoing release of GWAS data. By applying large-scale GWAS data, MR enables to strengthen the causal inference between blood metabolites and IAs with reverse causality prevented. However, we didn't explore the molecular mechanism involved in the contribution of metabolites to IAs and ruptures. Further mechanistic investigation would be appreciated.

In conclusion, we investigated the potential causal relationship between peripheral circulating proteins and metabolites and IAs. Our findings demonstrate that metabolic palmitoylcarnitine, stearoylcarnitine and 2-tetradecenoylcarnitine are causally linked to the formation and rupture of IAs. Hypertension partially mediates the causal effects Supplementary information.

## Author Contributions

L.Z. and X.L. conceived and designed experiments; data acquiring and analyzing were performed by X.L; K.X., Y.W. and L.Z. wrote the manuscript; all authors contribute to reviewing and editing.

## Ethical approval

According to the original GWAS protocols, informed consents were obtained from all participants, and all necessary ethical approvals for the GWAS were secured by the original GWAS authors.

## Availability of data and materials

GWAS data of blood metabolites, plasma proteins and intracranial aneurysms (the Bakker et al. GWAS cohort) and The FinnGen cohort are available in the corresponding publications [[15], [16], [17]] and website of https://www.finngen.fi/en. Hypertension GWAS is available on https://www.finngen.fi/en. We used R v.4.3.1 (https://www.r-project.org/), Two-sample MR v.0.5.7 (https://mrcieu.github.io/TwoSampleMR/).

## Declaration of competing interest

The authors declare that they have no known competing financial interests or personal relationships that could have appeared to influence the work reported in this paper.

## Figures and Tables

**Figure S1:**